# Acclimatization of Photosynthetic Apparatus of Tor Grass (*Brachypodium pinnatum*) during Expansion

**DOI:** 10.1371/journal.pone.0156201

**Published:** 2016-06-08

**Authors:** Wojciech Bąba, Hazem M. Kalaji, Agnieszka Kompała-Bąba, Vasilij Goltsev

**Affiliations:** 1 Department of Plant Ecology, Institute of Botany, Jagiellonian University, Lubicz 46, 31–512, Kraków, Poland; 2 Department of Plant Physiology, Warsaw University of Life Sciences SGGW, Nowoursynowska 159, 02–776, Warsaw, Poland; 3 Department of Botany and Nature Protection, University of Silesia, Jagiellonska 28, 40–032, Katowice, Poland; 4 Department of Biophysics and Radiobiology, Faculty of Biology, St. Kliment Ohridski University of Sofia, 8 Dr. Tzankov Blvd., 1164, Sofia, Bulgaria; Mount Allison University, CANADA

## Abstract

The aim of this study was to understand the acclimatization mechanisms of photosynthetic apparatus in *Brachypodium pinnatum* (L.) P. Beauv grass during its expansion. Twelve populations differentiated by age: young (30–50 years old), intermediate age (ca. 100 y) and old (>300 y) were studied. It was confirmed that the decrease of the number of genotypes as a result of environmental stress and competition were reflected in changes in chlorophyll fluorescence (ChlF) parameters. The old stands were dominated by a few genotypes which seem to be the best acclimatized to the self-shading/competition by lowering their photosynthetic performance during light-phase of photosynthesis. On the other hand, the 'high-speed' photosynthetic rate observed in the young populations can be seen as acclimatization to very adverse conditions. Our results clearly confirm that ChlF is a powerful method of inferring physiological mechanisms of the expansion of tor grass. The Principal Component and Redundancy Analyses, followed with k-means classification, allowed to find the differentiation of groups of distinct ChlF parameters and enabled us to relate them to changes in genotypic diversity of populations. We conclude that the plastic morphological and physiological response to changeable habitat light conditions with its optimum in half-shade refers to its forest-steppe origin.

## Introduction

Native species, similar to invasive alien (IAS) ones, can also have negative ecological and economic impact. They spread within their natural range attaining in some cases extreme abundances and exert effect on native vegetation [1]. *Brachypodium pinnatum* (L.) P. Beauv. belongs to the 'native invaders', which naturally occur in low abundance within calcareous grasslands. Its expansion, however, heavily reduces the biodiversity of calcareous grasslands [2,3] and often ends with formation of nearly monodominant stands [4]. The spread of false brome in Europe was triggered at the end of the 1960s as a result of the abandonment of traditional land use such as grazing and mowing [5,6]. Deposition of airborne nitrogen and phosphorus is considered to be a second possible reason for its expansion in Western Europe [7,8]. Numerous aspects of biology have been reported to date including the plastic response to light conditions [8], biomass and nutrient allocation, nutrient cycling [9–11], mycorrhizal colonization [12] karyotype evolution [13] and the population genetic diversity of this species along colonization gradients or under different management [6,14,15]. Moreover, several attempts have been made to control the *Brachypodium* expansion [16,17]. However, there is still a need for a quick, easy-to-perform and low cost method of monitoring the viability of large numbers of individuals, aimed at assessing its physiological acclimatization to changes in environmental conditions during expansion It is crucial in elaborating the proper management schemes.

Chlorophyll fluorescence (ChlF) measurements, which have been frequently performed [18–20], could be a method that meets these requirements and enables to assess different stress effects on photosynthetic apparatus using this technique, particularly on Photosystem II (PSII) status and linear electron transport rate. ChlF is a naturally occurring phenomenon, characteristic to all photosynthetic organisms. The ca 1–8% of the sun’s energy that is not used to drive photosynthesis is dissipated as heat radiation or re-emitted as light photons [21]. The analysis based on high time-resolution measurements of the ChlF transient represents a method for gaining detailed information about PSII photochemical activity, electron transport events and the different regulatory processes. Fast ChlF kinetics data are derived from the time dependent increase in fluorescence intensity achieved after application of bright light to a dark adapted sample. The resulting curve is called the Kautsky curve or ChlF transient [19].

The fluorescence parameters obtained in this way, called OJIP-test, allow the quantification of the stepwise flow of energy through PSII, using input data from the fluorescence transient and are formulated with a simplified model of the energy fluxes incorporating the parameters that define each type of flux [22,23]. The energy fluxes consist of an absorbed flux (ABS), trapping flux (TR), electron transport flux (ET) and the flux defining the dissipation of non-trapped energy as heat (DI)–a flux quantifying the reduction of Photosystem I (PSI) end acceptor (RE) was later introduced [19,24–26].

The aim of this research was to understand the mechanisms of acclimatization of photosynthetic apparatus during expansion of tor grass populations. Particularly, the following questions were asked: (i) How the ChlF parameters, related to different aspects of PSII functioning, change with habitat age and (ii) is there any relationship between genotypic/genetic diversity and ChlF parameters. It was expected that ChlF parameters can be used in a quick assessment of genotype acclimatization and can be helpful in management planning intended to prevent the future expansion of this species.

## Materials and Methods

### Species

*Brachypodium pinnatum* (L.) P. Beauv (subsequently referred to as *Brachypodium*) is a rhizomatous, perennial grass with widespread distribution in temperate regions of Northern Hemisphere. It forms clones, usually up to 1.5 (max 4) m in diameter with ca. 80% short rhizomes (1–10 mm) and dense tillers in form of 'clumps of shoots' (3–5 cm diameter) connected with longer rhizomes (100–200 mm) [15]. This enables it the long-term occupation of a given area and quick lateral spread. *Brachypodium* is both a diploid (2n = 18) and allotetraploid species (2n = 28) [27,28]. The stem height varies from 35 to 120 cm. It starts flowering at the end of June and set seeds from late July to September. The mean seed production on the study area varied from 2 to 45 per inflorescence and from 600 to 5000 per 1 m^2^ and was lowest on the old grasslands.

### Study area

The study area lies in Cracow-Częstochowa Upland, southern Poland. It consists of the system of the Upper Jurassic limestone valleys and hills on the plateau between them. The annual average temperature is 7.5°C in vegetation period and mean annual precipitation reaches 773 mm. *Brachypodium* occurs on the S, SW and SE slopes, and sometimes forms nearly monodominant stands [15,29]. 12 *Brachypodium*-dominated calcareous grasslands were selected for establishment of the study plots (250 m^2^ 50 x 50 m). The sites were classified according to grassland age and genotypic diversity of *Brachypodium* stands. Based on cadastral maps (1791–1970) and aerial photographs (1957–2009), the sites were classified as **young–** 30–50 years old: Racławice [19°40′ 37.06′′E, 50°12′ 02.10′′N], Powroźnikowa 1 [19°40′08.48′′E, 50°11′44.06′′N], Powroźnikowa 2 [19°40′04.53′′E, 50° 12′02.39′′N], Willisowe Skały [19°42′30.76′′E, 50°11′23.49′′N], **interm**ediate age–ca. 100 years old: Dolina Kobylańska [19°45′42.06′′E, 50°9′27.33′′N], Skala Żytnia [19°47′54.96′′E, 50°11′14.72′′N], WielkieSkały [19°48′20.17′E 50°11′23.49′′N], Bolechowice [19°46′58.08′′E, 50°9′11.28′′N], and **old**>300 years: Grodzisko [19°49′46.55′′E, 50°13′36.39′′N], Grodzisko-Onobrychis [19°49′36.86′′E, 50°13′40.54′′N], Dolina Będkowska [19°44′21.28′′E, 50°8′49.44′′N] and Dolina Kluczwody [19°49′7.04′′E, 50°9′49.94′′N]. Moreover, according to the results of the previous population genetic analyses based on AFLP markers, it was possible to relate age to genotypic richness (*G*) and the percentage of polymorphic loci (*PPL*), which decreased with the habitat age [6,15]. **The other criteria for classification** of *Brachypodium* populations were the differences in genotypic diversity and morphological parameters of its clones. According to the results of previous population genetic analyses based on AFLP markers, it was possible to relate age to genotypic richness (*G*) and the percentage of polymorphic loci (*PPL*), which both decreased with the habitat age [6,15]. Moreover, the average shoots and 'clumps of shoots' density/1m^2^[9],the number of leaves in clumps of shoots/1m^2,^ (given in S1–S3 Figs), roots and rhizome dry biomass **increased with habitat age** S4 and S5 Figs). In contrast, the mean density of generative shoots/1 m^2^ (S6 Fig) and the average seed production/1m^2^ [15] were lowest on the old grasslands. We found this as a sign of a competitive exclusion among genotypes during the *Brachypodium* expansion [15]. The Authors confirmed that the field studies did not involved endangered or protected species. The sampling sites were located on areas with no specific permissions required for locations and activities.

### Climatic data

The climatic data: monthly precipitation and mean air temperatures for young, intermediate and old populations were assessed on the basis of climatic service, http://klimat.icm.edu.pl/serv_climate.php (S1 Table).

### Soil and plant analyses

Five soil samples were collected from 0–30 horizons at each site for analysis of the physicochemical parameters. The soils samples were pooled, mixed, air-dried, and then grounded and sieved through 2 mm mesh. The particle size fractions (sand, silt and clay) were determined by sieving and sedimentation method (Prószyński method). Soil pH was measured electrometrically in water suspension following extraction with 1 M KCl and H_2_O (1:2.5). Organic matter content was estimated as loss on ignition (LOI) at 550°C of soil samples dried at 105°C for 12 h and expressed as a percentage of dry weight. Total N content was assessed applying the Kjeldahl method using Automatic Kjeldahl Digestion Units and UDK 129 Kjeldahl DKL Distillation Unit (VELP Scientifica, Italy). The total content of Ca, Mg, Mn, Cu and Fe in the soil and plant material was determined by flame atomic absorption spectrometry (Varian Spectra AA 330) after hot digestion of 3 mg of soil in mixture of 65% HNO_3_ and 35%H_2_O_2_ (8:2) with ETHOS ONE microwave system (Milestone, S.r.L., Italy) and exchangeable forms of metals in soil were estimated in 0.5 M HCl soil extracts [30].

### Leaf morpho-anatomical traits

Fully developed leaf blades from each of the populations were collected randomly in order to compare the morpho-anatomical traits among individuals of *Brachypodium* from the old and young populations. The leaf width (LW), height (LH), leaf area (LA), specific leaf area (SLA) were measured according to standard methods [31]. Anatomical measurements of leaves were done on slides, prepared following the standard method for fluorescence microscopy. Cross-sections of leaves were analyzed with Nikon Eclipse with a DS-Fi1-U2 camera (Ni-U, Nikon Co., Japan) to acquire microscopic images at 10× 20× magnification. The thickness of the leaf blades, width, height and area of the central vascular bundle of leaf, number of the sclerenchyma strands on the adaxial and abaxial sides of the tiller leaf were compared among the leaves from old and young populations.

### Measurement of Chl *a* fluorescence

The first fully developed leaves were collected at each location on July 2014, the optimum period for vegetative development of the *Brachypodium* [8]. The sampling was performed between 6–11 a.m. on cloudy days. 100 fully developed leaves per site were collected from randomly chosen plants. The leaves were put into the paper envelopes, sealed, placed in cooler bags and transported immediately to the laboratory. ChlF measurements were performed on the middle part of abaxial leaf blades away from the main leaf vein after additional dark adaptation (30 min) in a dark room using leaf clips. Fluorescence measurements were performed with the PocketPEA fluorimeter (Hansatech Instruments, King's Lynn, Norfolk, UK). For induction of fluorescence red actinic light was used (wavelength at peak 650 nm; spectral line half-width 22 nm) with the intensity of 3500 μmol m –^2^ s –^1^, and 1 second of transient fluorescence was recorded [26,32–34]. The fluorescence signal was collected with a maximum frequency of 10^5^ points s –^1^ (each 10 μs) within 0–0.3 ms, after which the frequency of recording gradually decreased, collecting a total of 118 points within 1 s. ChlF transient data were used to calculate basic parameters and the parameters needed for the OJIP-test (Table 1). The F_O_ level was measured as the fluorescence at 50 μs. The collected data were used for the calculation of basic parameters, while the fluorescence intensities determined at O-50 μs, J-2ms, I-30 ms and maximum fluorescence, P ~ 300 ms (F_M_)were used for the calculation of the OJIP test parameters (Table 1)[19,35,36]. To visualize the K and L bands, the collected data points were double normalized as relative variable fluorescence between points O-I, O-J and O-K [37]. Then, the kinetic differences between the old grasslands (treated as control) vs. interm and young were calculated (Table 1). This procedure helped to reveal bands that are normally hidden between the O and P steps on relative variable fluorescence (Table 1) [38].

**Table 1:** Summary of measured and calculated Chl *a* fluorescence parameters.

Fluorescence parameter	Description
**Measured parameters and basic JIP-test parameters derived from the OJIP transient**^**2**^^**,**^ ^**3**^^**,**^ ^**4**^ ^**7**^^**,**^ ^**11**^^**,**^ ^**12**^	
**F**_**O**_**~ F**_**50 μs**_	Minimum fluorescence, when all PSII RCs are open. Fluorescence intensity at 50 μs
**F**_**K**_	Fluorescence intensity at K-step (300 μs)
**F**_**J**_	Fluorescence intensity at the J-step (2 ms)
**F**_**M**_	Maximum recorded fluorescence at P-step (~300 ms)
**F**_**V**_ **= F**_**M**_**−F**_**O**_	Maximum variable fluorescence
**S**_**M**_ **= A**_**M**_**/(F**_**M**_**−F**_**O**_**)**	Standardized area above the fluorescence curve between F_O_ and F_M_ is proportional to the pool size of the electron acceptors Q_A_ on the reducing side of Photosystem II
**V**_**K**_ **= (F**_**300μs**_**−F**_**O**_**)/(F**_**M**_**−F**_**O**_**)**	Relative variable fluorescence at K-step (300 μs, K-band)
**V**_**I**_ **= (F**_**30ms**_**−F**_**O**_**)/(F**_**M**_**−F**_**O**_**)**	Relative variable fluorescence at I-step (30ms)
**M**_**0**_ **= 4 (F**_**300μs**_**−F**_**O**_**)/(F**_**M**_**−F**_**O**_**) = ΔV/Δt**_**0**_ **= TR**_**0**_**/RC–ET**_**0**_**/RC**	Approximated initial slope of the fluorescent transient. This parameter is related to rate of closure of reaction centers
**Specific energy fluxes expressed per active PSII reaction center (RC)** ^**2**^^**,**^ ^**3**^^**,**^ ^**5**^^**,**^ ^**8**^^**,**^ ^**11**^^**,**^ ^**12**^	
**ABS/RC = M**_**0**_**×(1/V**_**J**_**) ×[1 –(F**_**O**_**/F**_**M**_**)]**	Apparent antenna size of active PSII RC
**RC/CS**_**0**_ **= Fo x φ**_**Po**_ **x V**_**J**_**/M**_**0**_	Density of RCs (Q_A_ reducing PS II reaction centres)
**TR**_**0**_**/RC = M**_**0**_**×(1/V**_**J**_**)**	Trapping flux leading to Q_A_ reduction per RC
**ET**_**0**_**/RC = M**_0_*(1/V_**J**_)*ψ_**0**_	Electron transport flux per reaction center (RC) at t = 0
**DI**_**0**_**/RC = (ABS/RC)–(TR**_**0**_**/RC)**	Dissipated energy flux per reaction center (RC) at t = 0
**RE**_**0**_**/RC = M**_**0**_**(1/V**_**J**_**)(1 –V**_**J**_	Quantum yield of electron transport from Q_A_^−^to the PSI end electron acceptors
**N = (S**_**M**_**/S**_**S**_**) = Sm/M**_**0**_**×(1/V**_**J**_**), whereS**_**S**_ **= V**_**J**_**/M**_**0**_	the number indicating how many times Q_A_ is reduced while fluorescence reaches its maximal value (number of Q_A_ redox turnovers until F_M_ is reached); S_S_—normalized curve above O-J curve.
**Quantum yields and probabilities**^**2**^^**,**^ ^**3**^^**,**^ ^**5**^^**,**^ ^**7**^^**,**^ ^**8**^^**,**^ ^**11**^^**,**^ ^**12**^	
**φ**_**Po**_ **≡ TR**_**0**_**/ABS = [1 –F**_**O**_**/F**_**M**_**)] = F**_**V**_**/F**_**M**_	Maximum quantum yield of primary PSII photochemistry
**φ**_**Eo**_ **= (1 –F**_**J**_**/F**_**M**_**)(1 –V**_**J**_**)**	Quantum yield for electron transport from Q_A_^−^to plastoquinone
**φ**_**Do**_ **= F**_**O**_**/F**_**M**_	Quantum yield (at t = 0) of energy dissipation
**ψ**_**o**_ **(= ψ**_**Eo**_**) ≡ET**_**0**_**/TR**_**0**_ **= 1 –V**_**J**_	Probability (at time 0) that a trapped exciton moves an electron into the electron transport chain beyond Q_A_^–^
**φ**_**Ro**_ **= (1 –F**_**I**_**/F**_**M**_**)(1 –V**_**J**_**)**	Quantum yield for reduction of end electron acceptors at the PSI acceptor side (RE)
**γ**_**RC**_	Probability, that PSII chlorophyll molecule function as RC
**Performance indexes and driving forces**^**2**^^**,**^ ^**3**^^**,**^ ^**6**^^**,**^ ^**11**^^**,**^ ^**12**^	
**PI**_**ABS**_ **= γ**_**RC/**_**(1 – γ**_**RC**_**) ×φ**_**Po**_**/(1 – φ**_**Po**_**) ×ψ**_**o**_**/(1 –ψ**_**o**_**)**	Performance index of PSII based to absorption
**PI**_**total**_ **= PI**_**ABS**_**× δ**_**Ro**_**/(1 –δ**_**Ro**_**), whereδ**_**Ro**_ **= (1 –V**_**J**_**)/(1 –V**_**I**_**)**	Performance index he performance of electron flux to the final PSI electron acceptors
**Connectivity among PSII units**^**4**^^**,**^ ^**8**^^**,**^ ^**10**^^**,**^^**11**^	
**W = (F**_**100μs**_**−F**_**50μs**_**)/(F**_**2ms**_**−F**_**50μs**_**)**	Relative variable fluorescence in 100 s
**W**_**E**_ **= 1 –[(F**_**2ms**_**−F**_**300μs**_**) /(F**_**2ms**_**−F**_**50μs**_**)]**^**1/5**^	Model-derived value of relative variable fluorescence in 100 ms calculated for unconnected PSII units
**C = (W**_**E**_**−W)/[V**_**J**_**× W×(1 –W**_**E**_**)]**	Curvature constant of initial phase of the O-J curve
**P**_**G**_ **= F**_**O**_ **× C/(F**_**M**_**−F**_**O**_**)**	Probability of connectivity among PSII units (grouping probability)
**p = [P**_**2G**_**× (F**_**M**_**/ F**_**50μs**_**–1)]/[1 +P**_**2G**_**× (F**_**M**_**/ F**_**50μs**_**–1)]**	Connectivity parameter
**Differences in relative variable fluorescence between young, interm. age and old populations**^**11**^^**,**^^**12**^	
**W**_**OI**_ **= (F**_**t**_**−F**_**O**_**)/(F**_**30ms**_**−F**_**O**_**)**	Double normalized fluorescence readings at points O-I
**W**_**OJ**_ **= (F**_**t**_**−F**_**O**_**)/(F**_**2ms**_**−F**_**O**_**)**	Double normalized fluorescence readings at points O-J
**W**_**OK**_ **= (F**_**t**_**−F**_**O**_**)/(F**_**300μs**_**−F**_**O**_**)**	Double normalized fluorescence readings at points O-K
**ΔW**_**OI**_ **= W**_**OIyoung or interm**_**−W**_**OIold (control)**_	Differences in relative variable fluorescence at points O-I between young, intermediate age and old (control) populations
**ΔW**_**OJ**_ **= W**_**OJyoung or interm**_**−W**_**OJold (control)**_	Differences in relative variable fluorescence at points O-J between young, intermediate age and old (control) populations
**ΔW**_**OK**_ **= W**_**OKyoung or interm**_**−W**_**OKold (control)**_	Differences in relative variable fluorescence at points O-K between young, intermediate age and old (control) populations

Based on

^1^Malkin and Kok 1966

^2^Strasser et al. 1995

^3^Strasser et al. 2000

^4^Strasser and Stirbet 2001

^5^Strasser et al. 2004

^6^Tsimilli-Michael and Strasser 2008

^7^Strasser et al. 2010

^8^Stirbet and Govindjee 2011

^9^Brestic et al. 2012

^10^Stirbet 2013

^11^Zivcak et al. 2014

^12^Kalaji et al. 2014a

### Determination of Chl *a* and *b* content

The leaf greenness index as the average of five readings for each of 30 leaves per site was obtained using a portable chlorophyll meter (SPAD-502 Konica-Minolta, Japan). The SPAD recordings were obtained from a leaf disc of area 169.72 mm^2.^ After recording, the plant tissue was stored in a glass tube containing 5 mL DMSO (96% Dimethyl sulfoxide) [39]. The test tubes were incubated at 70°C for 48 hours. After cooling the extract in the dark, 3 mL aliquot was analyzed spectrophotometrically at 470, 647 and 663 nm wavelength with the DR 5000 spectrophotometer (Hach Lange). The chlorophyll *a* (Chl *a*) and *b* (Chl *b*) content was determined according to the following formulas [40]:

$$Chla=(12.25\times A_{663}-2.79\times A_{647})\times D$$ (1)

$$Chl\;b=(21.50\times A_{647}-5.10\times A_{663})\times D,$$ (2)

where A is the absorbance of wavelength, after the correction for scattering at 750 nm and *D* is the optical thickness of the cuvette [41]. Then, the chlorophyll content per unit leaf area (mg per m^2^) was calculated.

### Statistical analyses

The values shown in the tables represent the mean of (i) all fluorescence parameters, (ii) the values obtained in OJIP test, (iii) the content of selected mineral compounds in the soil and in the plants and (iv) leaf traits, were calculated for all habitat age. The differences in all these variables among the habitats of different age were tested with one-way ANOVA. Prior to the analyses, the assumption of ANOVA: (i) homogeneity and (ii) normality, symmetry of distribution and outliers were visually assessed and tested with (i) Levenne and (ii) QQ plots, boxplots and Shapiro-Wilk W tests [42]. The Welch ANOVA was used in the case of violation of homogeneity assumption. If the F-test was significant, a pairwise comparison of means was calculated using Tukey's test [42]. To visualize the divergence in overall changes of Chl *a* fluorescence traits among the grasslands of different age, a Principal Component Analysis (PCA) was performed. To relate the fluorescence parameters to genetic diversity and leaf Chl content, redundancy analysis (RDA) was performed. Genetic diversity: percentage of polymorphic loci (*PPL*), number of genotypes (*G*) and distribution of frequency of genotypes (Pareto, *beta*), was assessed using AFLP markers (details in Bąba *et al*. [6,15]. The Pareto index was high when many genotypes of comparable size occurred in population, and the lowest, when one of the few genotypes dominated the population. It could be used as a proxy value of competition among genotypes [43]. The significance of these variables was tested with the Monte Carlo permutation test (N = 999 permutations). In order to reveal the pattern of changes of ChlF parameters during expansion of *Brachypodium*, the classification of Chl *a* fluorescence parameters was performed with k-means clustering. The optimal number of groups was estimated based on the Calinski-Harabasz criterion [44]. All the statistical calculations were performed with R 3.2.0 packages MASS, *stats*, *agricolae* and *vegan* [45].

## Results

### Soil conditions

In field experiments, as opposed to laboratory ones, the crucial thing is the careful selection of study locations to make them as comparable as possible in terms of all factors, beside the ones of interest (i.e. chlorophyll fluorescence). The sites under study did not differ significantly in soil physico-chemical parameters: skeleton, soil particle fractions percentage content, soil reaction and most of micro- and macroelements content. It was only a slight (but not significant) increase in total or exchangeable forms of K, P, Fe, Cu and decrease in total Mg with grassland age (Table 2). Additionally, the mean content of selected macro—and microelements in the *Brachypodium* tissue did not show any pattern (Table 2).

**Table 2:** The physico-chemical parameters of soil and plant tissue concentration of selected micro- and macroelements.

	old grassland	intermediate grassland	young grassland
	(n = 4)	(n = 4)	(n = 4)
	Mean ± SD	Mean ± SD	Mean ± SD
**Soil physico-chemical property:**			
**Skeleton (g/100 g)**	32.72 ± 13.15	28.24 ± 13.07	30.51 ± 26.79
**Sand (%)**	18.50 ± 5.97	17.67 ± 4.16	15.0 ± 3.96
**Silt (%)**	60.00 ± 3.00	63.67 ± 5.03	64.5 ± 3.45
**Clay (%)**	17.00 ± 6.21	18.67 ± 3.05	20.00 ± 2.45
**pH (KCl)**	6.81 ± 0.20	6.81 ± 0.39	6.78 ± 1.21
**N (%)**	0.43 ± 0.18	0.60 ± 0.33	0.35 ± 0.19
**SOM (%)**	14.01 ± 4.87	16.09 ± 7.14	12.39 ± 5.75
**Exchangeable Ca (mg kg**^**-1**^**)**	1718.05 ± 342.5	2754.57 ± 1123.3	3425.23 ± 765.4
**Exchangeable K (mg kg**^**-1**^**)**	381.7 ± 19.79	341.2 ± 22.77	273.2 ± 13.23
**Exchangeable P**_**2**_**O**_**5**_ **(mg kg**^**-1**^**)**	596.50 ± 296.93	316.70 ± 197.23	107.20 ± 55.18
**Exchangeable Mg (mg kg**^**-1**^**)**	644.00 ± 30.58	574.00 ± 40.01	667.50 ± 36.65
**Exchangeable Mn (mg kg**^**-1**^**)**	45.34 ± 23.3	39.45 ± 11.98	67.23 ± 23.7
**Exchangeable Cu (mg kg**^**-1**^**)**	2.6 ± 0.4	3.7 ± 0.5	3.9 ± 0.7
**Exchangeable Fe (mg kg**^**-1**^**)**	645.76 ± 342.64	785.34 ± 453.23	342.45 ± 231.34
**Exchangeable Zn (mg kg**^**-1**^**)**	34.07 ± 24.9	45.02 ± 35.2	38.34 ± 17.3
**Total Ca (mg kg**^**-1**^**)**	5738.07 ± 3577.41	10628.91 ± 8597.48	191333.45 ± 2698.13
**Total K (mg kg**^**-1**^**)**	951.54 ± 362.7	1171.12 ± 734.8	795.54 ± 496.5
**Total Mg (mg kg**^**-1**^**)**	5342.45 ± 2342.4	6456.34 ± 2453.5	6895.57 ± 4534.2
**Total Mn (mg kg**^**-1**^**)**	440.73 ± 118.29	1142.91 ± 706.30	598.41 ± 331.08
**Total Cu (mg kg**^**-1**^**)**	15.57 ± 4.29	21.10 ± 18.62	21.74 ± 7.65
**Total Fe (mg kg**^**-1**^**)**	13622.68 ± 925.04	17253.25 ± 4640.51	11702.65 ± 3995.32
**Total Zn (mg kg**^**-1**^**)**	304.77 ± 108.09	353.96 ± 203.51	332.50 ± 190.91
	(n = 30)	(n = 30)	(n = 30)
**Plant**			
**Ca (mg kg**^**-1**^**)**	23.78 ± 8.7	20.60 ± 12.7	19.86 ± 17.6
**K (mg kg**^**-1**^**)**	32.00 ± 5.7	39.30 ± 28.4	42.40 ± 15.7
**Mg (mg kg**^**-1**^**)**	2.21 ± 0.95	1.73 ± 1.02	1.60 ± 1.24
**Mn (mg kg**^**-1**^**)**	1.38 ± 0.53	1.34 ± 0.78	3.05 ± 1.54
**Cu (mg kg**^**-1**^**)**	0.20 ± 0.04	0.12 ± 0.09	0.11 ± 0.8
**Fe (mg kg**^**-1**^**)**	3.31 ± 0.87	8.55 ± 3.56	6.70 ± 3.98
**Zn (mg kg**^**-1**^**)**	0.90 ± 0.34	0.57 ± 0.24	1.19 ± 0.68

### Variation of morpho-anatomical and chemical characteristics of leaves of *Brachypodium*

The significant differences were found in morphometric traits of leaf blades during the expansion of *Brachypodium*. The plants from old populations had higher values of leaf dry mass (LDM), leaf area (LA), leaf length (LL) and leaf width (LW) than those which came from interm and young populations of *Brachypodium* (Table 3). Individuals from old populations had significantly higher number of leaves per ramet (= rooted shoot *sensu* Falińska *et al* [46]), than in the other populations (11.9±2.5 vs. 10.2±4.1 and 10.1±4.1, Welch ANOVA, F = 22.9, p<0.001). However, there were no significant differences in specific leaf area (SLA) among the studied populations.

**Table 3:** The leaf traits, leaf chlorophyll content and parameters of the Chl a fluorescence tor grass *Brachypodium pinnatum* from population of different age: young (30–50 years old), intermediate age (ca. 100 years) and old(> 300 years). **The means of for each populations are presented. and means±SE for the particular age classes were compared with ANOVA, followed with Tukey post-hoc test. Values with the same letters are not significantly different at p < 0.05 level. Abbreviations: LA—leaf area, LDM—Leaf dry matter content, SLA—specific leaf area, LL—**leaf length, LW—leaf width. **Other abbreviations in Table 1. Locality: Rac–Racławice, Pow1 –Powroznikowa 1, Pow2 –Powroznikowa 2, WilSk–Wilisowe Skały, Kob–Dolina Kobylanska, Żyt–Zytnia, WielSk–Wielskie Skały, Bolech–Bolechowice, Gro_Onob–Grodzisko Onobrychis, Bedk–Dolina Bedkowska, DKluc–Dolina Kluczwody, Gro–Grodzisko.**

Locality	Rac	Pow1	Pow2	WilSk	Kob	Żyt	WieSk	Bolech	Gro_Onob	Bedk	DKluc	Gro	Total		
age	young	young	young	young	interm	interm	interm	interm	old	old	old	old	young	interm	old
													mean	mean	mean
**n**	81	100	92	97	99	98	100	96	100	100	100	100			
**Leaf traits**															
**Leaf chlorophyll content**															
**Ch**_**tot**_^**A**^	307.52	272.27	241.16	245.31	404.23	428.69	376.94	362.25	455.32	397.05	448.44	433.05	266.56±17.6a	393.03±16.9b	433.36±15.0b
**Chl a**^**A**^	226.15	204.25	187.57	186.11	295.79	302.98	283.89	275.76	337.53	296.78	334.15	319.10	201.02±10.7a	289.60±7.0b	321.89±10.7b
**Chl b**^**A**^	81.37	70.01	53.59	59.19	108.51	125.71	93.04	86.27	117.79	100.23	114.28	113.94	66.04±7.1a	103.38±10.1b	111.56±4.5b
**Chl a/b**	2.78	2.92	3.50	3.14	2.73	2.41	3.05	3.20	2.86	2.96	2.92	2.80	3.04±0.1a	2.84±0.2b	2.88±0.04b
**SPAD**	24.44	23.7	23.8	24.6	24.5	27.5	28.5	23.2	34.7	33.9	35.01	36.1	24.88 a±7.6a	26.5 a±5.5a	34.59 b±5.6b
**SLA [g/mm**^**2**^**]**	12290.4	16056.5	15.776.1	13254.8	11853.67	13700.69	16362.86	11468.35	11290.4	15156.5	14.776.1	13254.8	14224.81±567.2	13609.73±453.4	13315.78±123.4
**LA [mm**^**2**^**]**	845.20	1019.24	713.45	811.23	789.23	1122.0	936.52	906.97	1614.78	1273.51	1581.16	1123.24	847.20±345.3a	930.73±435.2a	1527.05±231.3b
**LDM [g]**	0.070	0.062	0.069	0.074	0.059	0.068	0.076	0.063	0.129	0.09	0.117	0.113	0.063±0.05a	0.065±0.05a	0.115±0.03b
**LL[mm]**	151.27	183.35	222.94	150.84	189.78	290.36	242.14	217.51	335.43	390.06	320.82	274.6	197.55±98.5a	229.0±97.5a	310.19±57.4b
**LW[mm]**	6.69	4.22	4.94	5.06	6.13	5.43	8.10	4.78	8.11	8.67	7.72	6.82	5.93±1.5a	6.06±0.9a	7.82±0.5b
**Chlorophyll *a* fluorescence**															
**Measured parameters**															
**Fo** ^**B**^	7.6	5.59	7.79	7.34	5.35	6.15	6.6	7.34	8.57	6.94	7.1	8.29	7.08±1.36a	6.36+1.60b	7.72±1.25a
**F**_**K**_^**B**^	11.98	9.93	12.93	11.65	8.87	11.23	11.36	12.16	15.38	12.01	12.02	13.54	11.62±1.76a	10.90±2.75b	13.23±2.51c
**F**_**J**_^**B**^	15.18	15.68	16.59	14.93	14.44	17.13	18.7	18.16	24.06	19.32	17.82	19.76	15.59±2.36a	17.11±4.46b	20.24±3.77c
**Fm** ^**B**^	29.97	26.19	27.55	26.54	25.5	27.38	27.01	29.17	36.51	34.93	34.32	31.02	27.56±7.03a	27.26±5.19a	34.19±4.88b
**Fv** ^**B**^	22.37	20.6	19.76	19.2	20.15	21.24	20.4	21.83	27.94	27.99	27.22	22.73	20.48±5.35a	20.90±5.75a	26.47±4.53b
**Calculated parameters**															
**V**_**L**_	0.08	0.08	0.13	0.09	0.07	0.1	0.09	0.1	0.1	0.07	0.08	0.1	0.11±0.04a	0.09±0.03a	0.08±0.03b
**V**_**K**_	0.18	0.21	0.28	0.21	0.18	0.25	0.24	0.23	0.25	0.18	0.18	0.23	0.22±0.12a	0.22±0.05a	0.21±0.03a
**V**_**J**_	0.33	0.49	0.47	0.39	0.45	0.52	0.6	0.5	0.56	0.44	0.39	0.51	0.42±0.11a	0.52±0.1b	0.47±0.09c
**V**_**I**_	0.68	0.84	0.73	0.63	0.81	0.85	0.86	0.79	0.88	0.86	0.81	0.9	0.72±0.11a	0.83±0.05b	0.86±0.04c
**M**_**o**_	0.81	0.85	1.22	0.96	0.77	1.00	0.95	0.9	0.99	0.73	0.72	0.91	0.96±0.35a	0.91±0.27b	0.83±0.21c
**PI**_**abs**_	23.04	8.34	13.71	16.75	9.44	7.64	4.62	8.55	6.69	10.27	13.64	7.02	15.46±11.69a	7.56±3.72b	9.40±3.6c
**PI**_**total**_	21.08	6.28	21.38	32.64	7.58	6.41	4.52	11.62	4.70	5.71	9.45	3.68	19.24±13.56a	7.46±5.45b	5.91±1.92c
**Sm**	28.56	17.04	26.01	34.18	20.08	13.77	19.84	18.45	15.52	15.4	14.78	12.39	26.45±19.79a	18.03±4.98b	14.52±4.38c
**N**	99.52	32.11	92.19	114.06	37.66	28.03	34.18	38.36	29.37	27.92	29.86	23.92	84.47±34.18a	34.56±14.56b	27.77±7.28b
**RC/CS**_**0**_ ^**B**^	2.80	2.66	2.50	2.61	2.90	2.63	3.30	3.22	3.88	3.49	3.18	3.59	2.64±0.12a	3.01±0.30b	3.53±0.28b
**ABS/RC**	3.7	2.19	4.31	4.00	2.22	2.46	2.09	2.53	2.30	2.05	2.31	2.48	3.55±2.21a	2.32±0.71b	2.28±0.33b
**DI**_**o**_**/RC**	1.06	0.47	1.57	1.35	0.53	0.58	0.52	0.69	0.55	0.41	0.48	0.68	1.11±1.16a	0.58±0.37b	0.53±0.16b
**TR**_**o**_**/RC**	2.64	1.72	2.74	2.65	1.69	1.88	1.57	1.84	1.74	1.64	1.83	1.79	2.43±1.11a	1.74±0.37b	1.75±0.23b
**ET**_**o**_**/RC**	1.83	0.87	1.53	1.69	0.92	0.88	0.62	0.93	0.76	0.91	1.11	0.88	1.48±0.94a	0.84±0.27b	0.91±0.20b
**RE**_**o**_**/RC**	1.06	0.27	0.93	1.18	0.33	0.27	0.22	0.41	0.21	0.23	0.35	0.18	0.86±1.01a	0.31±0.18b	0.24±0.08b
**Yield/Efficiency**															
**φ**_**Po**_	0.74	0.79	0.69	0.71	0.78	0.77	0.75	0.74	0.76	0.8	0.79	0.72	0.73±0.08a	0.76±0.04b	0.77±0.05b
**ψ**_**o**_	0.67	0.51	0.53	0.61	0.55	0.48	0.4	0.5	0.44	0.56	0.61	0.49	0.58±0.11a	0.48±0.1b	0.52±0.09a
**φ**_**Eo**_	0.49	0.4	0.37	0.43	0.43	0.37	0.3	0.37	0.34	0.44	0.48	0.35	0.42±0.09a	0.36±0.09b	0.40±0.08a
**δ**_**Ro**_	0.47	0.31	0.48	0.59	0.35	0.31	0.37	0.41	0.29	0.26	0.31	0.22	0.46±0.23a	0.36±0.09b	0.27±0.08c
**φ**_**Ro**_	0.23	0.13	0.18	0.25	0.15	0.12	0.11	0.16	0.09	0.11	0.15	0.07	0.19±0.1a	0.13±0.04b	0.10±0.04c
**φ**_**Do**_	0.26	0.21	0.31	0.29	0.22	0.23	0.25	0.26	0.24	0.2	0.21	0.28	0.27±0.08a	0.24±0.04b	0.23±0.05b
**γ**_**RC**_	0.76	0.68	0.78	0.76	0.68	0.7	0.67	0.71	0.69	0.67	0.7	0.71	0.74±0.08a	0.69±0.05b	0.69±0.03b
**Connectivity among PSII units**															
***W***	0.124	0.081	0.123	0.124	0.088	0.092	0.07	0.093	0.093	0.077	0.093	0.081	0.113±0.03a	0.085±0.03b	0.086±0.01b
***C***	1.121	0.881	0.756	0.714	0.598	0.589	0.664	0.608	0.526	0.739	0.651	0.585	0.868±0.41a	0.614±0.17b	0.625±0.15b
***P***_**G**_	0.288	0.281	0.192	0.423	0.163	0.184	0.196	0.188	0.199	0.182	0.17	0.165	0.296±0.18a	0.182±0.06b	0.179±0.04b
***P***	0.585	0.576	0.675	0.674	0.541	0.557	0.51	0.544	0.505	0.622	0.621	0.526	0.628±0.11a	0.538±0.09b	0.568±0.09b

^A^ mg/m^2^

^B^ [a.u. x 1000]

**Fig 1:**
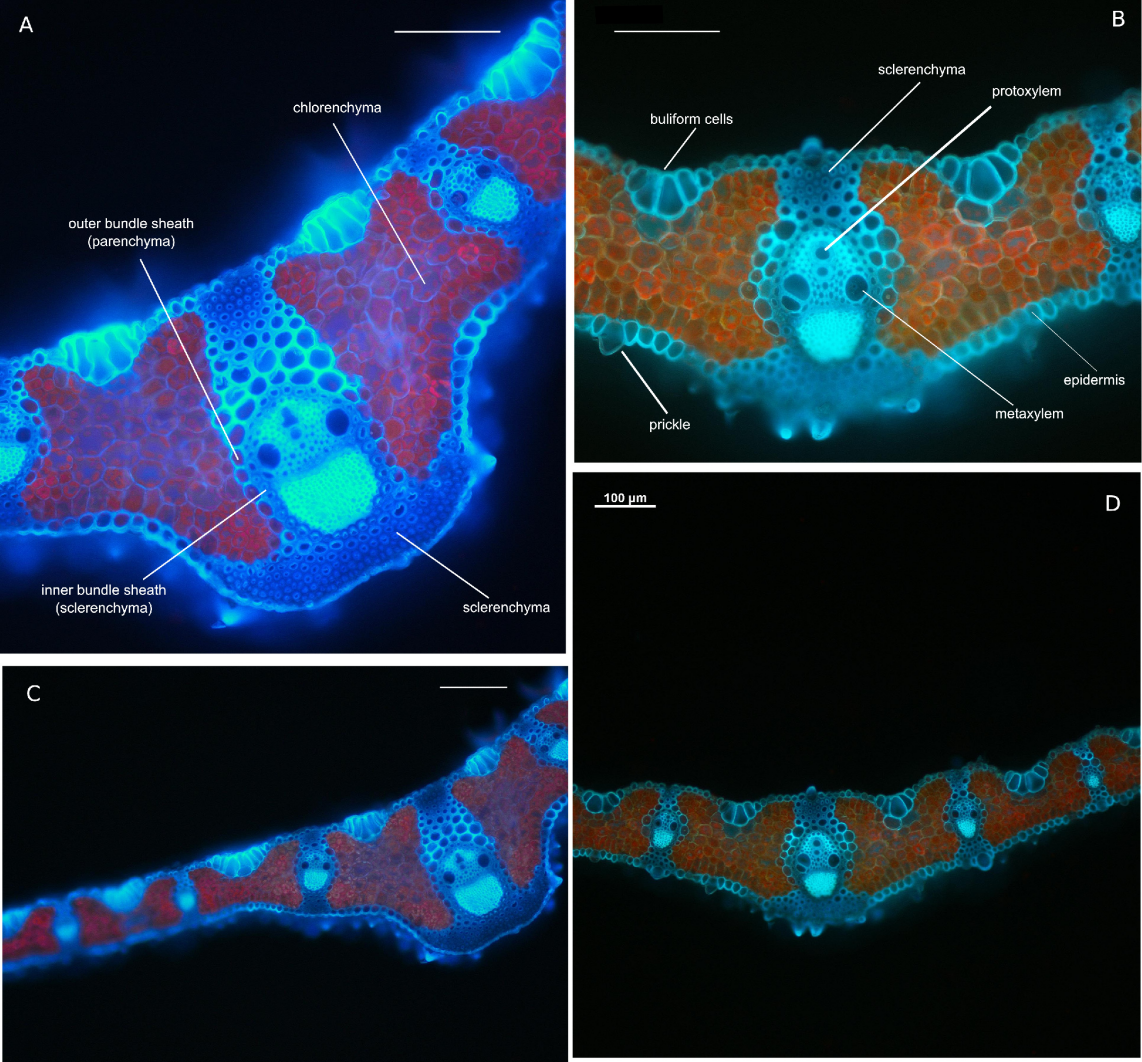
*Brachypodium pinnatum* leaf anatomy from fluorescence microscopy. Transverse cross section of leaf blade of individuals from old (A, C) and young (B, D) populations. Notice the differences in number and size of bulliform cells occurred on the adaxial side of leaf blade. Transverse section in the midrib at median level (A, B). The differences in (i) thickness and shape of the leaf blades in zone of the central rib, width of the central rib of tiller leaf, (ii) surface, height and width of the central vascular bundle and(iii) number of the sclerenchyma strands on abaxial side of leaf and (iv) distribution of are visible. Detailed leaf measurements were presented in the Results. A, B—20× magnification, C, D—10× magnification. Bars on each of the pictures indicate 100 μm.

The mean thickness of the tiller leaf blades of plants from young populations and width of central rib were lower than those from old ones (224 vs. 269 μm and 170 vs. 238μm respectively; Fig 1). Moreover, the differences were found in width (84 vs. 114μm) height (123 vs. 129μm) and area (8574 vs 10050μm^2^) of the central vascular bundle of leaf, the number and shape of bulliform cells (6–8 vs. 5–6), the number of the sclerenchyma strands on adaxial (5–7 vs. 4–5) and abaxial (4–5 vs. 6–7) sides of the tiller leaf when compared among the leaves of individuals from old and young populations (Fig 1). The chloroplasts in the inner part of mesophyll aligned in vertical columns along the plant cell walls, which is a well-known mechanism of avoidance of photodamage in plants [47]; Fig 1). However, the distribution of chloroplasts in leaves from young populations was more uniform across mesophyll, while those from old ones had bigger chloroplasts located close to the upper and lower epidermis (Fig 1).

Leaves of individuals from old and intermediate age populations had significantly higher Chl *a*, and Chl *b* content, than young ones. However, it was only slightly higher Chl *a*/*b* ratio in young populations (3.04 vs. 2.84 in interm and 2.88 in old populations respectively; Table 3). The increase in total leaf Chl content with population age was also reflected in pattern of leaf greenness index measured by SPAD (Table 3).

### Chl *a* fluorescence changes along habitat age gradient

**Fig 2:**
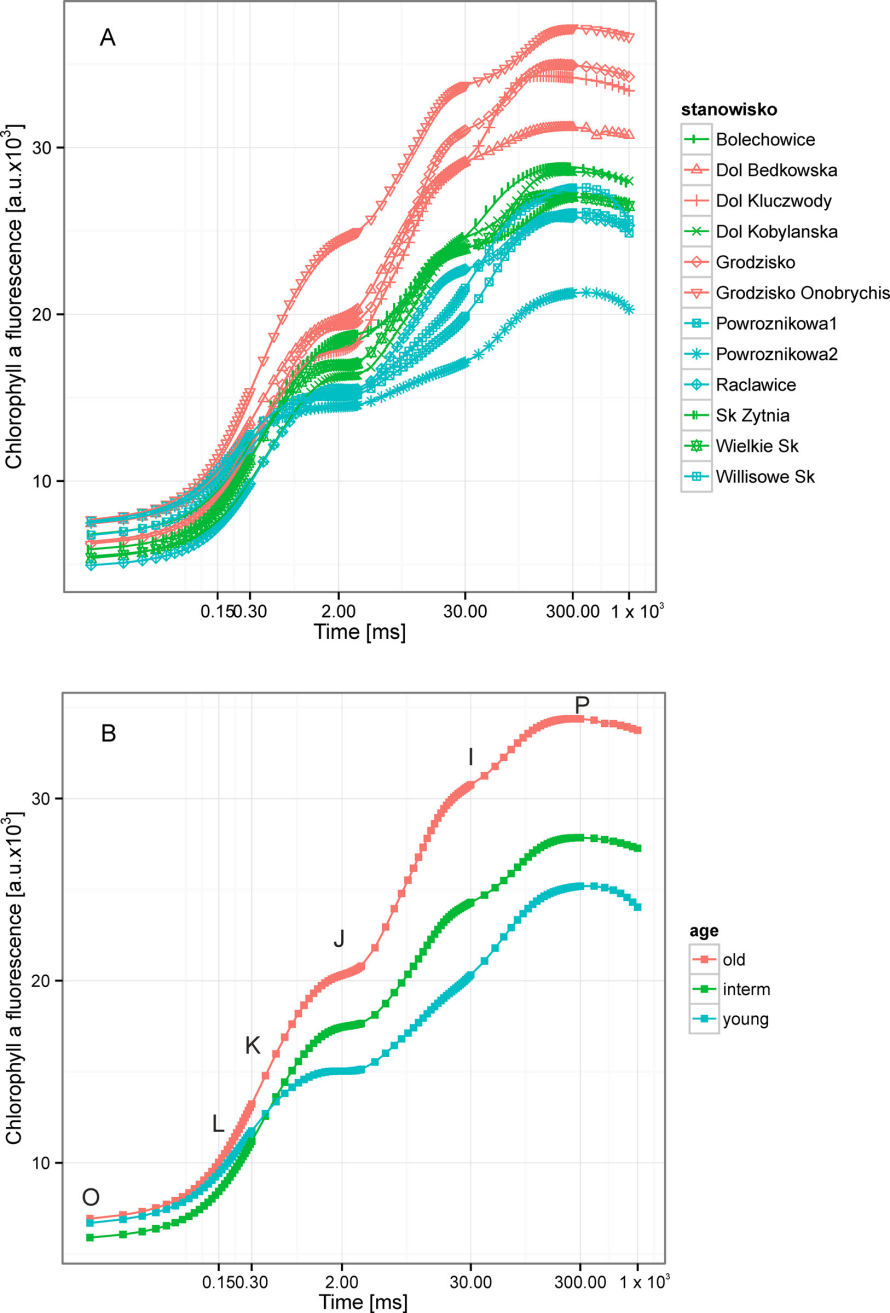
The chlorophyll fluorescence (ChlF) transients from dark-adapted leaves of expansive grass *Brachypodium pinnatum* from young (30–50 years), intermediate age (ca. 100 years) and old (>300 years) calcareous grasslands. The results were plotted on logarithmic scale from F_O_(50 μs) to 1 s. A. The fluorescence curves for all 12 populations under study divided into age classes. The time points important for the calculation of JIP test were marked: O–fluorescence intensity recorded at F_O_ (50 μs), L–at 150 μs, K–at 300 μs J–2 ms, I–at 30 ms, P–maximum fluorescence intensity (F_M_) at ca. 1 s. B. The curves of average ChlF values for each age classes.

The apparent differences in *Brachypodium* leaf traits were reflected in the state of Photosystem II, measured by ChlF. The mean prompt ChlF (OJIP) curves both for particular populations and for populations of different age differed both in the shape and amplitude, especially among the young vs. interm and old populations (Fig 2). This suggests possible differences in energy fluxes at the donor as well as at the acceptor side of PSII [23,41]. Moreover, it was also confirmed by mean values of measured and calculated fluorescence parameters (Table 3). Significant differences in formal characteristics of ChlF rise were found: F_O_, was significantly lower in interm populations, F_K_ and F_J_and its values increased from young to old populations.

**Fig 3:**
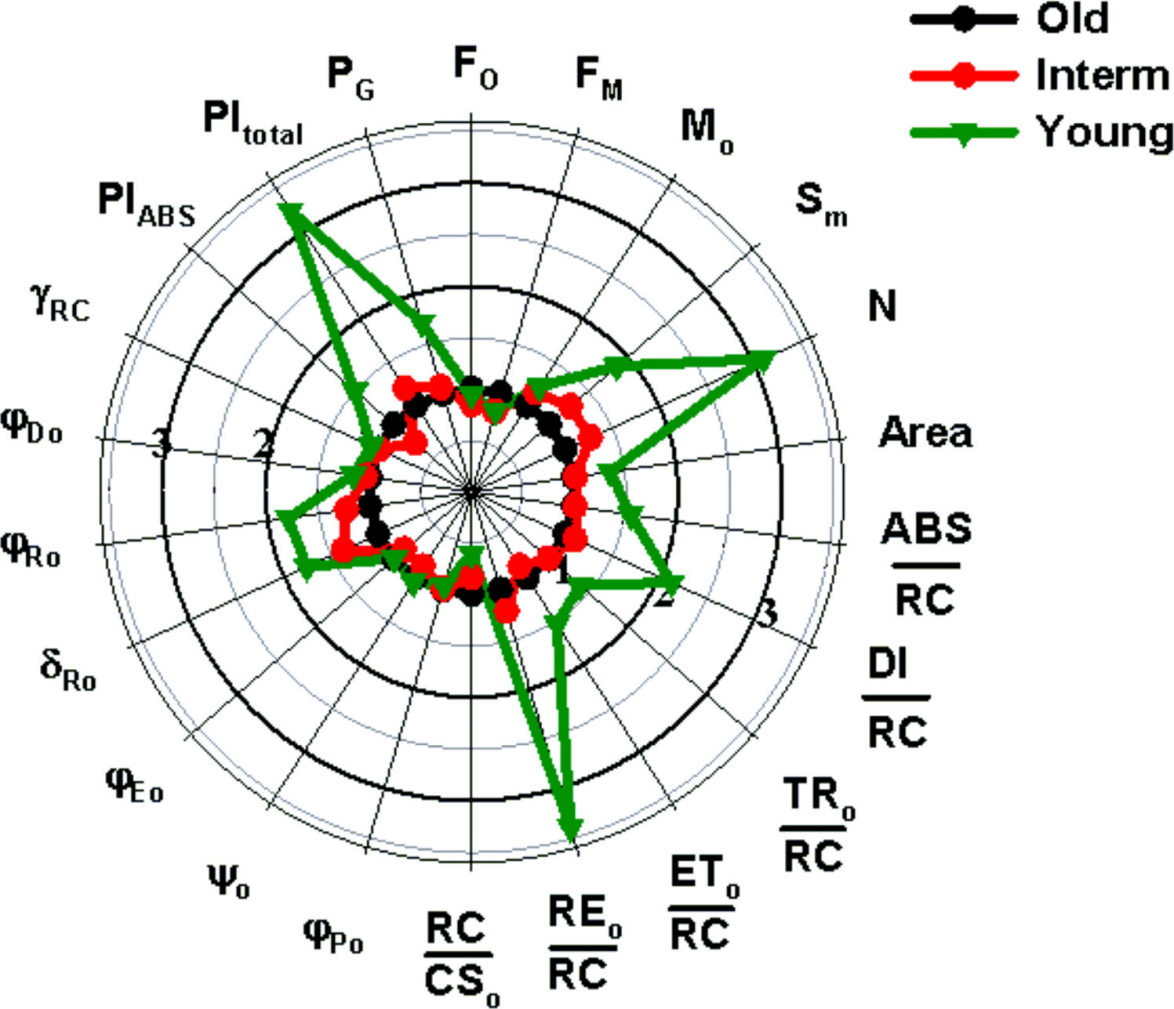
Variation in parameters reflecting morphological, chemical and photosynthetic conversions in PSII in leaves of *Brachypodium* plants from population with different age. The values of each parameter are normalized to values corresponding parameter from old-age population.

The fluorescence rise at the OJ phase was light dependent and provided the information about antenna size and connectivity of PSII reaction centers [48]. The significant peak on the double-normalized fluorescence curve was observed at J (V_J_) in young populations(Table 3). It was more pronounced when the course of the Fl rise was expressed as a difference between the analyzed curves. As a referent curve, the normalized fluorescence transient for plants from old populations was accepted as it was assumed that it as an end-point on the expansion gradient (Fig 3).

**Fig 4:**
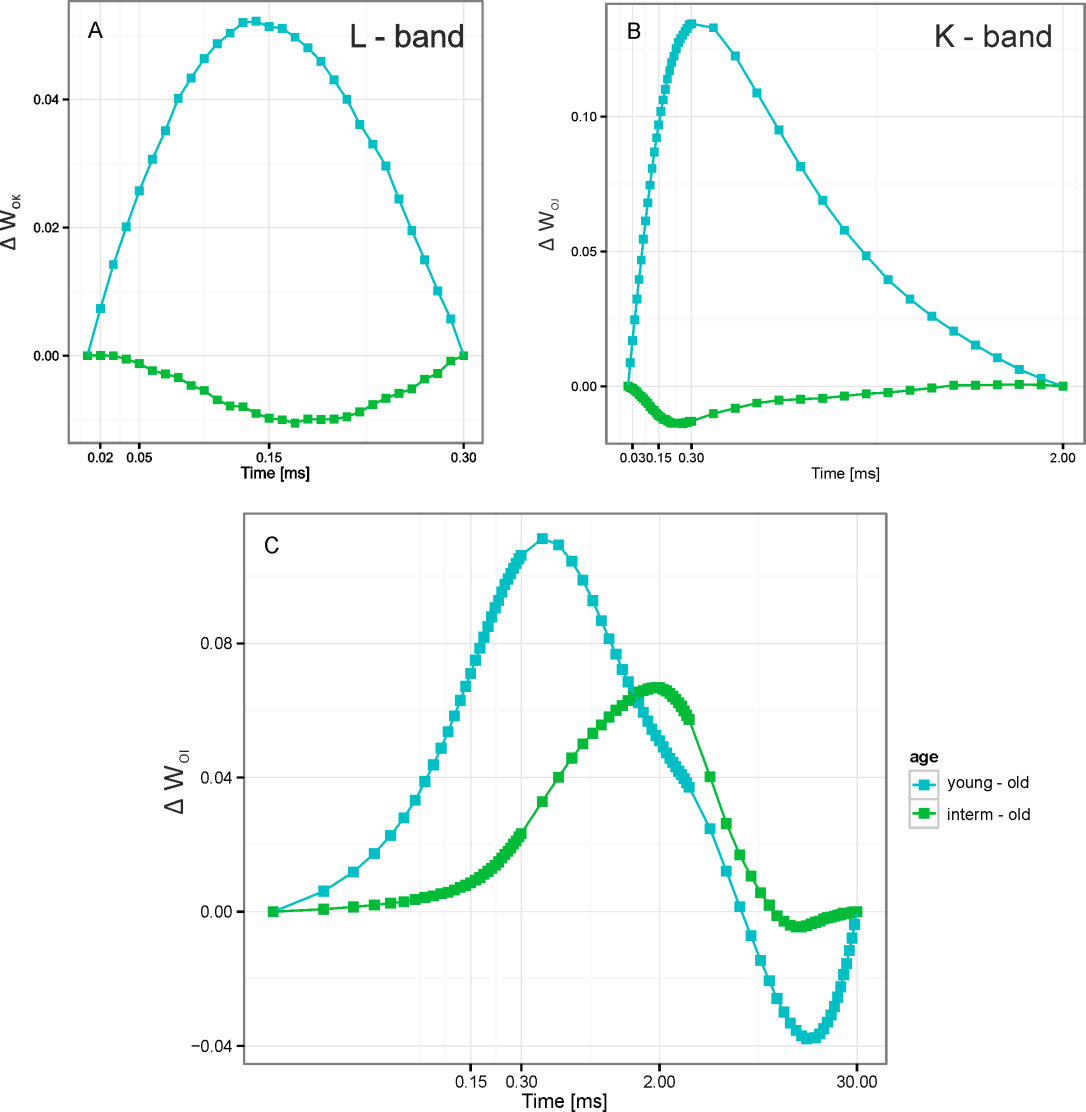
The chlorophyll fluorescence transients from dark-adapted leaves of expansive grass *Brachypodium pinnatum*. A. ChlF curves represent the increase of the relative variable fluorescence in young and intermediate age population relative to old ones (treated as control) between points F_O_ and F_K_; ΔW_OK_ = (F_t_-F_O_)/(F_K_-F_O_); L–band. B. ChlF curves represent the increase of the relative variable fluorescence in young and intermediate age population relative to old (control) ones between points F_O_ and F_J_ (ΔW_OJ_ = (F_t_-F_O_)/(F_J_-F_O_); K-band, C. The curves represent the increase of the relative variable fluorescence in young and intermediate age population relative to old (control) ones between points F_O_ and F_I_ (ΔW_OI_ = (F_t_-F_O_)/(F_I_-F_O_).

This indicated a slight limitation of electron transport from Q_A_ to Q_B_ as indicated by the values 1-V_J_, a probability of trapped PSII electron transfer from reduced Q_A_ to Q_B_ [41]. The double normalizations of fluorescence curves enabled us also to reveal the less pronounced positive L (V_L_) and K (V_K_) bands of fluorescence rise in young and interm populations as compared to old ones (Table 3, Fig 4). If the curves were normalized at O and I points (Fig 4) the main two bands appeared in “young–old” curve reflecting the well pronounced J band and well expressed K peak. For the “interm–old” differential curve the K peak at 0.3 ms became dominant. Separately, the effect of the population age on L—and K-bands could be better expressed in the differential curves at double normalization at time intervals “0.02–0.3 ms” and “0.02–2.0 ms”, respectively (see Fig 4).

Increasing positive peaks in these time regions in plants from interm and from young populations as compare to old population reflected more ungrouped PSII units in photosynthetic membranes and less activity of PSII oxygen evolving complex (OEC). The J–P rise of the prompt ChlF curve was attributed to the thermal phase of the fluorescence transient. It reflected a reduction in the electron transport chain [49,50] and, represented the electron transport from Q_A_ beyond PSI. The V_I_ values increased significantly with age of populations (Table 3).

The average values of maximum quantum efficiency of PSII φ_Po_ for all the populations under study was lower than typical for healthy plants in all populations (ca 0.83)[51] and ranged from 0.73 to 0.77 and were slightly, but significantly reduced in the young populations (Table 3; Fig 2; Fig 3). Moreover, the maximum fluorescence value F_M_, and the variable part of the ChlF, F_v_ were highest in old populations (Table 3; Fig 2; Fig 3). The parameters, reflecting the size of electron acceptor pools available on the reducing side of Photosystem II (S_M_)and at PSI acceptors (N) were lowest in the old populations. Moreover, the significant decrease in the standardized area (S_M_) was observed with age of population (Table 3; Fig 2; Fig 3).

The value of RC/CS_0_ (reflecting the relative density of active PSII reaction centers) was significantly lower (of about 40%) in plants from young as compare to old populations. In contrast of this, all parameters presenting the energy fluxes in one active reaction center (ABS/RC, TR_0_/RC, ET_0_/RC, RE_0_/RC and DI/RC) were visibly increased in the young populations. This increase was especially expressed for parameters correlation with the electron transfer site within PSI–from PQH_2_ to PSI end acceptors: RE_0_/RC, φ_Ro_ and δ_Ro_ (Fig 3; Table 3). The most sensitive to the population age parameter was the total Performance Index (PI_total_), which combines the efficiency of energy conversion both in PSII and PSI, significantly decreased with age of populations.

**Fig 5:**
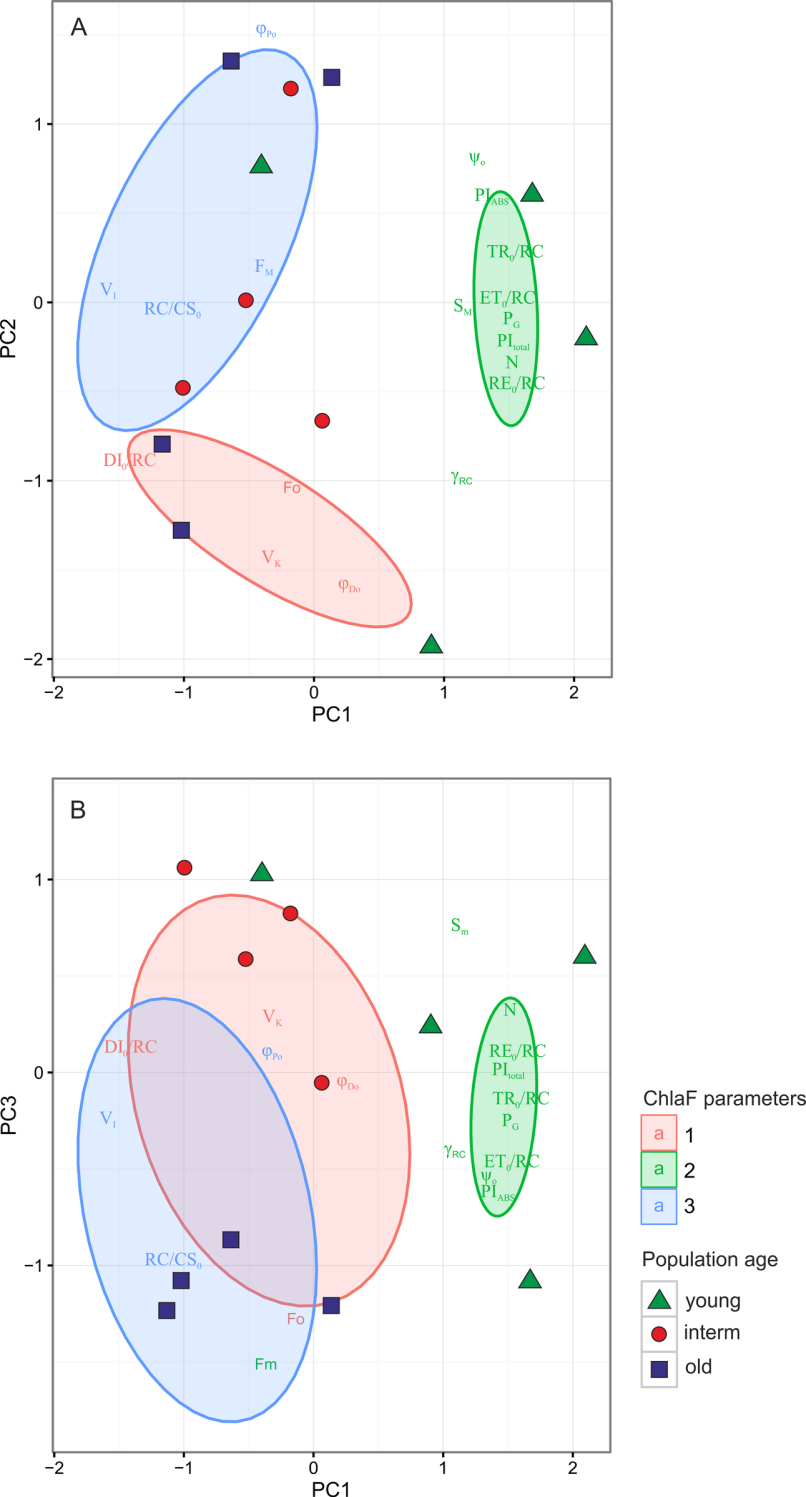
Principal Component (PCA) ordination diagram displaying the relationships between the Chl *a* fluorescence measurements and parameters. The classification of Chl *a* fluorescence parameters were performed with k-means clustering. The optimal number of groups (3) was estimated based on Caliński-Harabasz criterion. The result of classification was superimposed onto the ordination diagram. The ellipses represent 95% confidence intervals for the groups

The Principal Component Analysis (PCA, Fig 5; Table 4, S2 Table) was used in order to reduce the multivariate fluorescence data into the few principal components and to find the pattern of changes in fluorescence parameters during the expansion of *Brachypodium*. The classification of the ChlF parameters with the k-means method, based on the Calinski-Harabasz criterion, revealed n = 3 optimal number of groups, which were separated by the first and second PCA axes (Fig 5). The three first PCA axes explained 91.35% of variation in the dataset. The first axis explained 54.45% variation and clearly separated three of four young populations from the interm and old population with higher value of relative fluorescence at point I (V_I_). The PCA confirmed that the young populations were characterized by higher values of ChlF related to the plastoquinone (PQ) size pool (S_M_), higher electron transport rate and overall PSII performance (TR_0_/RC, ET_0_/RC,PI_ABS_, PI_total_), a higher number of Q_A_ turnover (N) and higher probability with which an electron from the intersystem carriers moves to reduce end electron acceptors at the PSI acceptor side (RE_0_/RC).

**Table 4:** The results of Principal Component Analysis (PCA) and Redundancy Analysis (RDA). The correlations of variables with the first three principal components (PC1-PC3, RDA1-RDA3) and cannonical correlations of leaf chlorophyll content and genetic diversity with Chl *a* fluorescence parameters were shown. Values ca. 0.7 are marked in bold.

**Parameters/Axes**	**PC1**	**PC2**	**PC3**
**Eigenvalues**	9.802	4.092	2.55
**Variation explained (%)**	54.45	22.73	14.16
**Cumulative proportion (%)**	54.45	77.19	91.35
**F**_**o**_ **~ F**_**50**_ **μs**	-0.083	-0.597	**-0.773**
**V**_**I**_	**-0.959**	0.047	-0.148
**V**_**K**_	-0.193	**-0.879**	0.179
**F**_**M**_	-0.222	0.123	**-0.915**
**N**	**0.902**	-0.219	0.220
**S**_**M**_	**0.695**	-0.015	0.464
**PI**_**total**_	**0.962**	-0.189	0.035
**PI**_**ABS**_	**0.829**	0.352	-0.373
**TR**_**0**_**/RC**	**0.971**	0.175	-0.128
**DI**_**0**_**/RC**	**-0.825**	-0.527	0.082
**ET**_**0**_**/RC**	**0.925**	0.015	-0.296
**RE**_**0**_**/RC**	**0.964**	-0.224	0.045
**RC/CS**_**0**_	**-0.656**	-0.020	-0.586
**ψ**_**o**_	**0.758**	0.492	-0.333
**φ**_**Po**_	-0.223	**0.921**	0.058
**φ**_**Do**_	0.177	**-0.963**	-0.032
**γ**_**RC**_	**0.688**	-0.598	-0.249
**P**_**G**_	**0.956**	-0.054	-0.149
**Canonical axes**	**RDA1**	**RDA2**	**RDA3**
**Eigenvalue**	5.020	4.679	2.582
**Variance explained (%)**	28.90	24.80	12.792
**Cumulative variance (%)**	28.90	53.70	66.49
**G**	**0.814**	-0.209	0.521
**PPL**	0.347	-0.363	0.342
**Pareto (beta) index**	**0.843**	-0.488	0.218
**Chl**_**tot**_	**-0.714**	-0.302	0.246
**Chl a/b ratio**	0.485	0.019	-0.309
**Residual variance**	0.335		

They also characterized with increase values of parameter related to the probability of grouping of PSII reaction centers (P_G_, Table 3; Fig 5). The second axis, which explained 22.73% variation in the data, separated the group of parameters related to the ChlF rise (V_K_) and quantum yield (at t = 0) of energy dissipation (φ_Do_), from the group of parameters related to fluorescence intensity F_M_, and maximum quantum efficiency of PSII (φ_Po_). The third PCA axis, which explained 14.16% of variation, separated the old populations with higher F_O_, F_M_ and RC/CS_0_values, from interm age populations (Fig 5).

**Fig 6:**
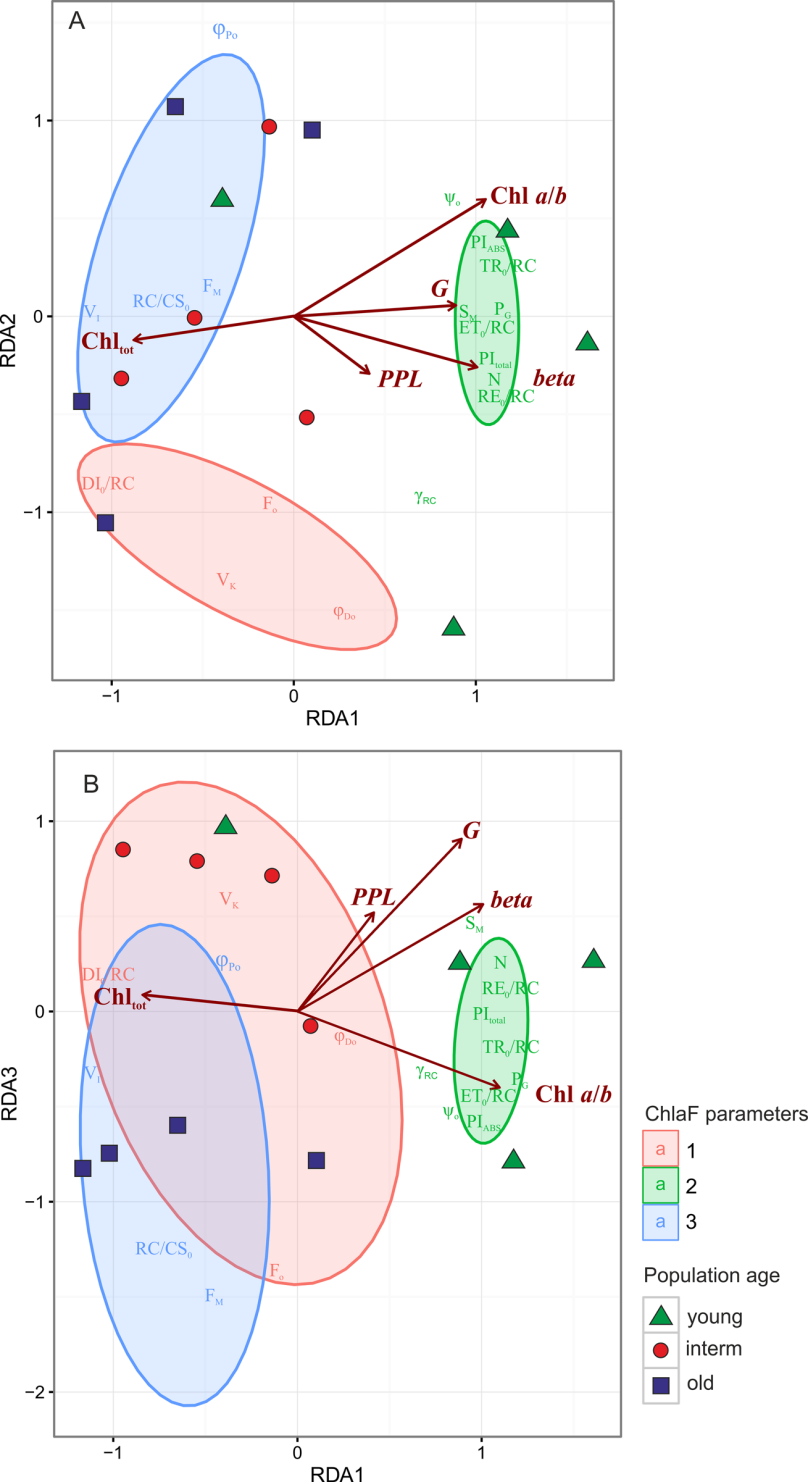
Redundancy Analysis (RDA) ordination diagram displaying the relationships between the (i) Chl *a* fluorescence measurements and parameters and (ii) genetic, genotypic diversity and chlorophyll content in leaves. The first two RDA axes explained of 28.9 and 24.8% of variation in the data respectively. The classification of Chl *a* fluorescence parameters were performed with k-means clustering. The optimal number of groups (3) was estimated based on Caliński-Harabasz criterion. The result of classification was superimposed onto the ordination diagram. The ellipses represent 95% confidence intervals for the groups.

**Fig 7:**
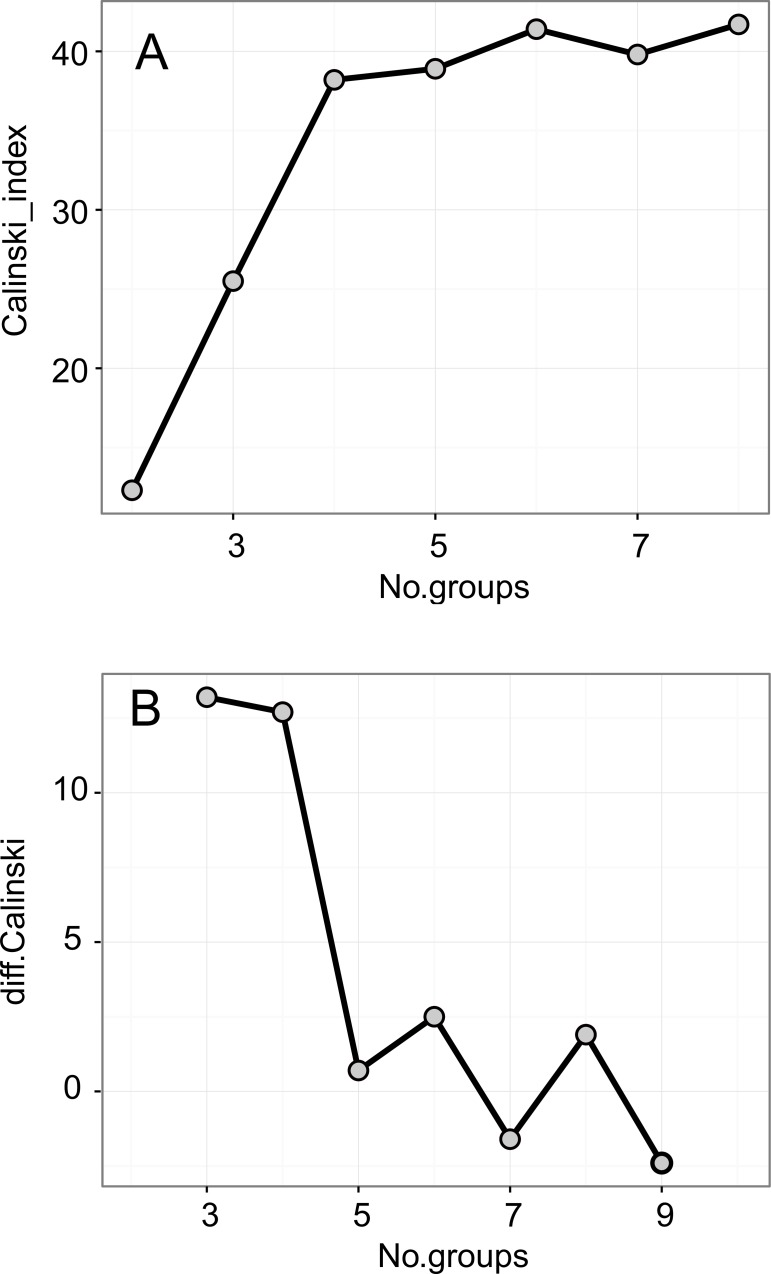
The optimal number of groups based on Calinski-Harabasz index. A. differences in Calinski-Harabasz index. B. The peak in 3b panel point 3 as a optimal number of groups.

Redundancy Analysis (RDA, Figs 6–7, and Tables 4 and 5) is a two-table method in which the gradient found in the fluorescence data, observed in PCA, could be directly related to the external variables. The first three RDA axes explained 66.5% variance in the fluorescence data (Table 4). The main gradient of variability along the first RDA axis, which explained 28.9% variation, could be related, as in PCA, to differences among young and other populations (Fig 6). The ChlF parameters recorded for young populations: lower activity of PSII oxygen evolving complex (OEC), lower values of maximum quantum efficiency of PSII (φ_Po_) and significantly reduced value RC/CS_0_, reflecting the relative density of active reaction centers, higher size of electron acceptor pools available on the reducing side of PSII (S_M_), between the both photosystems and at PSI acceptors (Area and N) and higher energy fluxes per one active reaction center are highly positively correlated to the value of the Pareto (*beta*) index high population genotypic diversity (*G*) and, weekly—percentage polymorphic (*PPL*) loci and Chl a/*b* ratio, (Table 5). This could be explained by populations of *Brachypodium* colonizing the new habitats, consisting of high numbers of equal size genotypes, characterized, on average, by high efficiency of PSII and high connectivity among PSII units. In the course of expansion some clones started to dominate others. They differed in structural traits expressed by higher Chl_tot_ content in leaves (Fig 6, Tables 4 and 5).

**Table 5:** Results of Redundancy Analysis (RDA) that relates selected genetic diversity (G—number of AFLP genotypes, Pareto *beta* index) and leaf chlorophyll content with chlorophyll fluorescence parameters. Pareto *beta* is index measuring the spatial distribution of genotypes and could be related to inter- and intraclonal competition. The highest value of *beta* were recorded in population with many equall-sized genotypes while the lowest with population dominated with few large clones. The results of Monte Carlo permutation test (with 999 permutations) of the variables is presented: Var—variance explained, F—pseudo-F, and significance level

Variable	Df	Var	F	Pr(>F)	
**G**	1	4.0038	2.87	0.03	*
**PPL**	1	0.5681	0.408	0.68	ns
**Pareto (beta)**	1	5.990	2.35	0.04	*
**Chltot**	1	7.221	2.88	0.014	*
**Chl a/b ratio**	1	1.537	1.22	0.065	ns
**Residual**	3				

* denotes statistically significant and ns—not significant results

The second RDA axes, which explained 24.8% variation in the data, differentiated groups of parameters related to the fluorescence rise from those related to maximal efficiency of PSII (φ_Po_). On the other hand, the third RDA axis, which explained 12.8%, separated the interm and old populations with similar efficiency of PSII but different F_O_, F_M_ and RC/CS_0_ values

## Discussion

Understanding of the processes related to establishment and expansion of species in the novel habitats is crucial to nature conservation, for example for development of management plans in order to protect the biodiversity of species-rich grasslands or preventing the colonization and spreading of invasive species. There is ample evidence that the probability of successful establishment in novel environment increases with the number of individuals in founder groups which come from different sources and with the number of repeated introductions [52–55]. However, many theoretical and empirical studies seem to indicate that founder effects and bottlenecks as a scenario of many colonizations, due to geographical separation of populations It have resulted in genetically uniform populations of many expansive species [54,56–59]. As confirmed previously by genetic (AFLP) analyses [15], the colonization of *Brachypodium pinnatum* follows the first model. It started from seeds from multiple locations and during the first 30–50 years it often produced monodominant stands especially on former fallow lands or on abandoned (i.e. not grazed or mowed) calcareous grasslands. At this stage, the grass formed stands consisted of many genotypes. On the intermediate and old grasslands (from 100 up to >300 years old), a strong decrease in the number of genotypes (*G*) and slight decrease in the percentage of polymorphic loci was observed (*PPL*)[6,15]. Although the data on (mostly neutral) genetic variability could be important for distinguishing 'individuals' of *Brachypodium* in the field conditions, there is the need for additional ecological or physiological data for the assessment of probability maintenance its viability or further expansion [55, 60]After establishment, individuals face a series of environmental stresses (high light, drought, high or low temperature and nutrient deficiency), inter- and intraspecific competition, introduced predators and novel or changed diseases [57]. The performed researches have revealed that the inter–and intraclonal competition and environmental conditions result in strong changes in the ecological properties of *Brachypodium* individuals and populations: increase in stem density per 1m^2^, clone diameter, lateral vegetative spread, mean number of leaves per ramet and strong decrease in seed production [15]. Moreover, during expansion, an increase in leaf length, leaf width, LA and overall leaf area are also observed. These changes are reflected in the leaf anatomy and showed a plastic acclimatization of *Brachypodium*to possibly high-light in young populations and high-light competition in old ones. The leaves of *Brachypodium* from young populations possesses traits typical for scleromorphic, sun leaves with big chloroplasts, distributed uniformly across mesophyll, while the other ones from old grasslands, additionally to strongly marked scleromorphism, display the optimal distribution of chloroplasts typical for leaves grown in dense stands [61]. The big chloroplasts are distributed close to the lower and upper epidermis.

Apart from changes in population, genotypic diversity and ecological traits during *Brachypodium* expansions, leaf structural changes and in ChlF parameters related to various aspects of PSII functioning were also visible. Our results confirmed the increase in Chl*a*, Chl*b*, total Chl content, and leaf greenness index (SPAD)with population age (Table 3). The values of Chl *a/b* ratio recorded in young populations were close to 3:1, often assumed as optimal to C3 plant [62], while slightly lowered in interm and old populations (2.84–2.88). Our results confirm those obtained by Fabell *et all* [63], Stroch *et all* [64] and Živčák *et al* [41], who observed relatively low differences in Chl *a/b* ratio in plants growing in high light and shade conditions. The changes in fast Chl *a* fluorescence kinetics could be related to photosynthesis performance since a light saturation point was found at ~ 1000μmol photons m^–2^ s^–2^ in young populations and ~ 1200 μmol photons m^–2^ s^–2^ in old ones (unpublished data—not shown). On the other hand, the increase in total Chl content in old populations is a typical reaction of shade leaves. Pattanayak *et al* [65] related this phenomenon to an increase in stand density, overall leaf area and possibly due to self-shadowing of leaves within *Brachypodium* stands. However, Murchie and Horton [66] and Murchie and Lawson [67] had found that in shade-grown plants there was no change in the Chl *a/b* ratio, while the Chl content decreased. Thus, the decrease in Chl *a/b* ratio in low light conditions does not seem to be a universal phenomenon, and the level of its dependence on light intensity strongly depends on plant species [41].

The size of the PSII antenna per active reaction center (ABS/RC) was also larger in young populations and these differences are preserved after correction for connectivity [41].The relative density of active PSII reaction centers (RC/CS_0_) is significantly lower (of about 40%) in plants from young populations compared to old ones. Hence, both pigment composition and prompt ChlF induction analysis indicate that *Brachypodium pinnatum* belongs to a group of plants with changeable antenna size [68]. Moreover, in this study, signs of reorganization of PSII units with population age were observed. The O-J part of prompt fluorescence kinetic curve is used to estimate the connectivity parameter among PSII units [23,26,69–71]. The calculated parameters associated with connectivity: probability of connectivity among PSII units–p [36], were 150% and 40% higher in leaves from young populations compared to the interm and old ones. The other connectivity parameters (P_G_) were also slightly higher in young populations (Table 3). The value of p parameter ranged between 0.53 to 0.67, which confirmed that the antenna organization of all populations studied followed the “connected unit” model, since the connectivity parameter p obtained ranged between 0 and 1 [69]. This means that the excitation energy of closed RCs can be transferred to a number of nearby open RCs [41].

Biotic stress affects plant growth through reduction of photosynthesis [72]. The initial slope of variable fluorescence M_0_, within rapid ChlF kinetics, indicates more rapid initial accumulation of closed RCs in leaves from young populations as compared to the interm and old ones. Moreover, the higher values of ChlF at the J and the I steps, and hence higher V_J_ and V_I_ values in the plants from old populations point to limited number of electron carriers on the PSII acceptor side [26,73]. Detailed analysis, based on the selected parameters (S_m_, N), suggests a decreased size of the pool of PSII and PSI electron carriers (from Q_A_ to ferredoxin), as well as, a decrease in the number of Q_A_ turnovers between F_O_ and F_M_. PSII is one of the most susceptible components of the photosynthetic machinery. Abiotic stresses, such as drought or high light, results in an over-reduction of the electron transport chain (ETC) [74–77]. The specific energy fluxes in one active reaction centers (ABS/RC, TR_0_/RC, ET_0_/RC, RE_0_/RC and DI_0_/RC) are visibly decreased with population age. This decrease is especially expressed for parameters correlation with the electron transfer site within PSI–from PQH_2_ to PSI end acceptors: RE_0_/RC, φ_Ro_ and δ_Ro._ These could be mechanisms by which the PSII of plants from old populations reduce the rate of electron transport chain by converting the excess of absorbed light into thermal energy. [26,78,79]. The diminished efficiency of each RC (PI_ABS_ and PI_total_) is compensated by an increased RC/CS_0_ (reflecting active reaction center density) per leaf area as well as by higher total Chl concentration in photosynthesizing tissue of plants from old populations.

The above mentioned results demonstrate the very plastic response of *Brachypodium* to changeable environmental conditions, reflected in reduction of the maximum quantum yield of PSII (φ_Po_) in the early stages of expansion while increasing the quantum yield and probability for electron transport from Q_A-_ to PQ (φ_Eo_; ψ_O_). In addition, the γ_Rc_ decrease in old and interm populations, caused a reduction in the amount of light harvesting complexes in PSII.

Among the ChlF parameters used in this study, the performance index (PI) provides the information on the general state of plants and their vitality [51]. It combines the information about the concentration of the fully active reaction center per chlorophyll, primary photochemistry and electron transport (Strasser *et al*. 2004). P_ABS_ is related to energy conservation of the photons absorbed by PSII in the form of reduced intersystem electron acceptors, and PI_total_ in the form of reduced acceptors of PS I. Changes in PI susceptible to changes in antenna properties, electron trapping efficiency and transport beyond Q_A_ [51]. Živčák *et al* [80] pointed out PI as very sensitive index to prolonged drought stress in winter wheat. In this study, significantly higher values of performance indices were found: higher by 50% for PI_ABS,_ and 300% for PI_total,_ in young populations.

## Conclusions

The results presented above confirm that the strong decrease of genotypes as a result of environmental stress occurs at the early and intermediate stages of *Brachypodium*expansion and is clearly reflected in changes in ChlF parameters. The old stands are dominated by a few genotypes which seem to be the best acclimatized to the self-shading/competition by lowering their photosynthetic performance during light-phase of photosynthesis. On the other hand the 'high-speed' photosynthetic observed in the young populations can be seen as acclimatization to very adverse conditions (probably combination of high light, high temperature, low nutrient water) in which the plants try to catch the sunflecks in the 'windows of opportunity' i.e. early morning or on cloudy days. The high capacity of *Brachypodium* for plastic morphological and physiological adjustment to changeable habitat light environment refers to its original forest-steppe coenological affinity [8]. Thus, the species has its physiological optimum in half-shade conditions of open forest from which it spreads on fallows or clearings.

The population genetic and ecological analyses are often costly, destructive to vegetation and time-consuming. The presented results clearly confirm that ChlF is a powerful method for inferring physiological mechanisms of expansion of tor grass. The PCA/RDA analyses followed with k-means classification based on the Calinski-Harabasz criterion allowed to distinguish groups of distinct ChlF parameters and allowed them to be related to the changes along stress-competition gradient, occurred during different stages of *Brachypodium* expansion.

## Supporting Information

S1 Fig Box and whiskers plots of average shoot density of *Brachypodium pinnatum*/1m^2^. The shoots were recorded on 20 1m^2^ plots on each *Brachypodium* populations. The values are averaged over three years: 2013–2015. On the charts the median (line inside the box), box (i.e. inter-quartile range, IQR) and whiskers, defined as 1.5*IQR, are presented. The points are the values beyond the norm (outliers).
(PDF)

S2 Fig Box and whiskers plots of average 'clumps of shoots' (see the Materials and Methods) density of *Brachypodium pinnatum*/1m^2^. The recordings were performed on 20 1m^2^ plots within each *Brachypodium* populations. The values are averaged over three years: 2013–2015. On the charts, the median (line inside the box), box (inter-quartile range, IQR) and whiskers (defined as 1.5*IQR) are presented. The points are the values beyond the norm (outliers).
(PDF)

S3 Fig Box and whiskers plots of average number of leaves in 'clumps of shoots' of *Brachypodium pinnatum*/1m^2^. The shoots were recorded on 20 1m^2^ plots within each *Brachypodium* populations. The values are averaged over three years: 2013–2015. On the charts, the median (line inside the box), box (inter-quartile range, IQR) and whiskers (defined as 1.5*IQR) are presented. The points are the values beyond the norm (outliers).
(PDF)

S4 Fig Box and whiskers plots of average rhizome dry biomass of *Brachypodium pinnatum*/1m^2^. in 2014, on each *Brachypodium* population 10 soil samples 20 x 20 x 20 cm were collected randomly within stands, and rhizomes were washed out. On the charts the median (line inside the box), box (inter-quartile range, IQR) and whiskers (defined as 1.5*IQR) are presented. The points are the values beyond the norm (outliers).
(PDF)

S5 Fig Box and whiskers plots of average root dry biomass of *Brachypodium pinnatum*/1m^2^. In 2014 on each *Brachypodium* population 10 soil samples 20 x 20 x 20 cm were collected randomly within stands, and roots were washed out. On the charts, the median (line inside the box), box (inter-quartile range, IQR) and whiskers (defined as 1.5*IQR) are presented. The points are the values beyond the norm (outliers).
(PDF)

S6 Fig Box and whiskers plots of average generative shoot density of *Brachypodium pinnatum*/1m^2^. The shoots were recorded on each *Brachypodium* populations on 20 1m^2^ plots. The values are averaged over three years: 2013–2015. On the charts the median (line inside the box), box (inter-quartile range, IQR) and whiskers (defined as 1.5*IQR) are presented. The points are the values beyond the norm (outliers).
(PDF)

S1 Table Monthly precipitation [mm] and mean air temperature[°C] in the year of the study. The values are averaged within age classes.
(DOCX)

S2 Table The results of Principal Component Analysis (PCA) and Redundancy Analysis (RDA). The percentage contributions of Chl of fluorescence parameters to the first three principal components (PC1-PC3) are shown.
(DOCX)

## Abbreviations

PSII: photosystem II
PSI: photosystem I
OEC: oxygen evolving complex
RC: reaction center
Chl: chlorophyll
ChlF: chlorophyll fluorescence
ABS: absorption flux
TR: electron trapping flux
ET: electron transport, Q_A_, primary PSII quinone acceptor
Q_B_: secondary PSII quinone acceptor
PQ: plastoquinone
PSI: PSII, Photosystem I, II
RC: reaction centers
OJIP: transient, fluorescence induction transient defined by the names of its intermediate steps
O-level: fluorescence level at 50 μs
J-level: fluorescence plateau at 2 ms
I-level: fluorescence plateau at 30 ms
P-level: the maximum fluorescence.
