# Supplementary figures and images for: Acclimatization of Photosynthetic Apparatus of Tor Grass (Brachypodium pinnatum) during Expansion
Source: PLoS One. 2016 Jun 8;11(6):e0156201. doi: 10.1371/journal.pone.0156201 (PMC4898706; doi:10.1371/journal.pone.0156201)

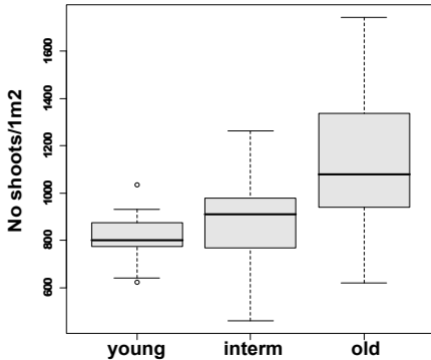

Supplement: S1 Fig — The shoots were recorded on 20 1m2 plots on each Brachypodium populations. The values are averaged over three years: 2013–2015. On the charts the median (line inside the box), box (i.e. inter-quartile range, IQR) and whiskers, defined as 1.5*IQR, are presented. The points are the values beyond the norm (outliers). (PDF) [file pone.0156201.s001.pdf]

No clumps of shoots/1m2

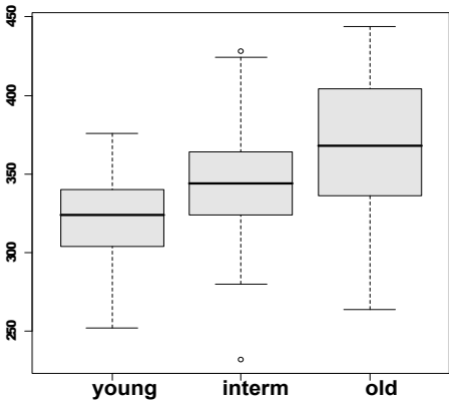

Supplement: S2 Fig — The recordings were performed on 20 1m2 plots within each Brachypodium populations. The values are averaged over three years: 2013–2015. On the charts, the median (line inside the box), box (inter-quartile range, IQR) and whiskers (defined as 1.5*IQR) are presented. The points are the values beyond the norm (outliers). (PDF) [file pone.0156201.s002.pdf]

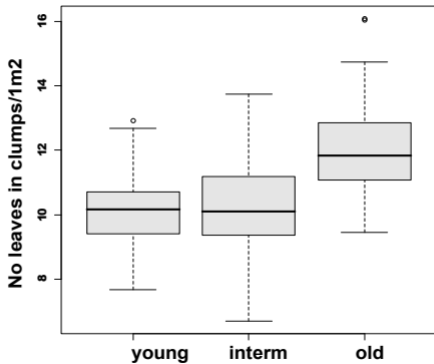

Supplement: S3 Fig — The shoots were recorded on 20 1m2 plots within each Brachypodium populations. The values are averaged over three years: 2013–2015. On the charts, the median (line inside the box), box (inter-quartile range, IQR) and whiskers (defined as 1.5*IQR) are presented. The points are the values beyond the norm (outliers). (PDF) [file pone.0156201.s003.pdf]

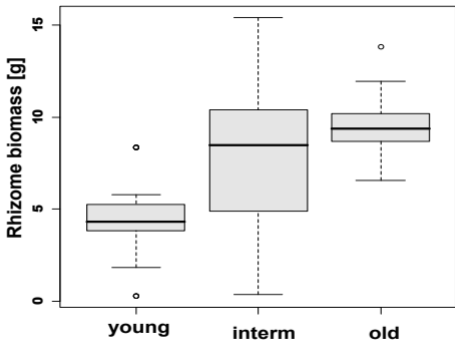

Supplement: S4 Fig — On the charts the median (line inside the box), box (inter-quartile range, IQR) and whiskers (defined as 1.5*IQR) are presented. The points are the values beyond the norm (outliers). (PDF) [file pone.0156201.s004.pdf]

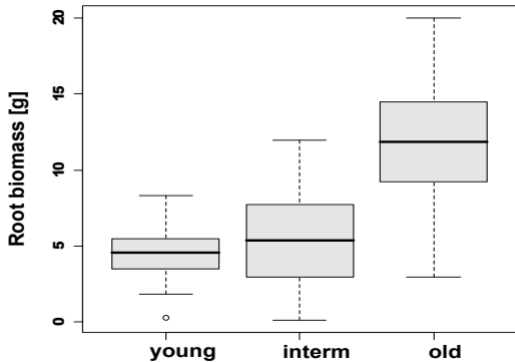

Supplement: S5 Fig — In 2014 on each Brachypodium population 10 soil samples 20 x 20 x 20 cm were collected randomly within stands, and roots were washed out. On the charts, the median (line inside the box), box (inter-quartile range, IQR) and whiskers (defined as 1.5*IQR) are presented. The points are the values beyond the norm (outliers). (PDF) [file pone.0156201.s005.pdf]

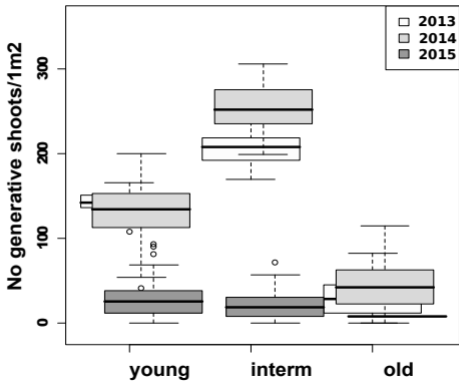

Supplement: S6 Fig — The shoots were recorded on each Brachypodium populations on 20 1m2 plots. The values are averaged over three years: 2013–2015. On the charts the median (line inside the box), box (inter-quartile range, IQR) and whiskers (defined as 1.5*IQR) are presented. The points are the values beyond the norm (outliers). (PDF) [file pone.0156201.s006.pdf]
