# Supplementary material for: Acclimatization of Photosynthetic Apparatus of Tor Grass (Brachypodium pinnatum) during Expansion
Source: PLoS One. 2016 Jun 8;11(6):e0156201. doi: 10.1371/journal.pone.0156201 (PMC4898706; doi:10.1371/journal.pone.0156201)
Supplement: S1 Table — The values are averaged within age classes. (DOCX) [file pone.0156201.s007.docx]

|  |  |  |  |  |  |  |  |  |  |
| --- | --- | --- | --- | --- | --- | --- | --- | --- | --- |
|  |  |  |  | Precipitation  (mm) |  |  | Temperature (ºC) |  |  |
|  | Year | Month | young | interm | old | young | interm | old |  |
|  | 2014 | 1 | 40.1 | 52.2 | 42.5 | -3.0 | -3.4 | -2,0 |  |
|  |  | 2 | 51.2 | 60.3 | 65.5 | 0.2 | -0.4 | 0,3 |  |
|  |  | 3 | 51.9 | 45.2 | 30.75 | 2.6 | 2.0 | 4,5 |  |
|  |  | 4 | 54.5 | 86.4 | 112.0 | 6.7 | 5.8 | 8,6 |  |
|  |  | 5 | 69.0 | 129.5 | 148.25 | 13.7 | 13.0 | 12,1 |  |
|  |  | 6 | 117.2 | 110.3 | 112.75 | 16.2 | 15.5 | 14,7 |  |
|  |  | 7 | 90.6 | 85.5 | 82.25 | 18.1 | 17.5 | 18,5 |  |
|  |  | 8 | 73.4 | 68.2 | 68.5 | 18.8 | 17.7 | 16,0 |  |
|  |  | 9 | 45.2 | 55.2 | 35.0 | 11.6 | 10.6 | 13,0 |  |
|  |  | 10 | 57.1 | 73.2 | 135.25 | 8.2 | 7.2 | 8,1 |  |
|  |  | 11 | 67.3 | 58.1 | 85.75 | 3.2 | 2.5 | 4,3 |  |
|  |  | 12 | 51.6 | 45.1 | 55.75 | -1.5 | -2.1 | -0,3 |  |
|  |  |  |  |  |  |  |  |  |  |
