# Supplementary material for: Acclimatization of Photosynthetic Apparatus of Tor Grass (Brachypodium pinnatum) during Expansion
Source: PLoS One. 2016 Jun 8;11(6):e0156201. doi: 10.1371/journal.pone.0156201 (PMC4898706; doi:10.1371/journal.pone.0156201)
Supplement: S2 Table — The percentage contributions of Chl of fluorescence parameters to the first three principal components (PC1-PC3) are shown. (DOCX) [file pone.0156201.s008.docx]

| **Parameters/Axes** | **PC1** | **PC2** | **PC3** |
| --- | --- | --- | --- |
| **F_o_ ~ F_50_ µs** | 0.071 | 8.723 | 23.461 |
| **V_I_** | 9.387 | 0.054 | 0.859 |
| **V_K_** | 0.379 | 18.887 | 1.258 |
| **F_M_** | 0.505 | 0.371 | 32.857 |
| **N** | 8.302 | 1.171 | 1.901 |
| **S_M_** | 4.928 | 0.006 | 8.455 |
| **PI_total_** | 9.434 | 0.870 | 0.049 |
| **PI_ABS_** | 7.019 | 3.029 | 5.454 |
| **TR_0_/RC** | 9.609 | 0.747 | 0.641 |
| **DI_0_/RC** | 6.936 | 6.786 | 0.261 |
| **ET_0_/RC** | 8.735 | 0.006 | 3.437 |
| **RE_0_/RC** | 9.473 | 1.226 | 0.081 |
| **RC/CS_0_** | 4.384 | 0.010 | 13.480 |
| **ψ_o_** | 5.862 | 5.913 | 4.346 |
| **φ_Po_** | 0.505 | 20.730 | 0.130 |
| **φ_Do_** | 0.320 | 22.676 | 0.039 |
| **γ_RC_** | 4.835 | 8.726 | 2.425 |
| **P_G_** | 9.316 | 0.070 | 0.868 |
